# Anticancer efficacy of the hypoxia‐activated prodrug evofosfamide (TH‐302) in osteolytic breast cancer murine models

**DOI:** 10.1002/cam4.599

**Published:** 2016-01-09

**Authors:** Vasilios Liapis, Irene Zinonos, Agatha Labrinidis, Shelley Hay, Vladimir Ponomarev, Vasilios Panagopoulos, Aneta Zysk, Mark DeNichilo, Wendy Ingman, Gerald J. Atkins, David M. Findlay, Andrew C. W. Zannettino, Andreas Evdokiou

**Affiliations:** ^1^ Discipline of SurgeryBreast Cancer Research UnitBasil Hetzel Institute and Centre for Personalised Cancer MedicineUniversity of Adelaide WoodvilleWoodvilleSouth AustraliaAustralia; ^2^ Department of RadiologyMemorial Sloan‐Kettering Cancer CenterNew YorkNew York; ^3^ Discipline of SurgerySchool of Medicine at The Queen Elizabeth HospitalUniversity of AdelaideWoodvilleSouth AustraliaAustralia; ^4^ Robinson Research InstituteUniversity of AdelaideAdelaideSouth AustraliaAustralia; ^5^ Discipline of Orthopaedics and TraumaUniversity of AdelaideAdelaideSouth AustraliaAustralia; ^6^ School of Medical SciencesMyeloma Research Laboratory Cancer ThemeSouth Australian Health and Medical Research Institute (SAHMRI)Faculty of Health ScienceUniversity of AdelaideAdelaideSouth AustraliaAustralia; ^7^ Labrinidis Adelaide Microscopythe Centre for Electron Microscopy and Microstructure AnalysisUniversity of AdelaideAdelaideSouth AustraliaAustralia

**Keywords:** Breast cancer, chemotherapy, evofosfamide, hypoxia, TH‐302, tumor growth

## Abstract

Tumor hypoxia is a major cause of treatment failure for a variety of malignancies. However, hypoxia offers treatment opportunities, exemplified by the development of compounds that target hypoxic regions within tumors. Evofosfamide (TH‐302) is a prodrug created by the conjugation of 2‐nitroimidazole to bromo‐isophosphoramide mustard (Br‐IPM). When evofosfamide is delivered to hypoxic regions, the DNA cross‐linking effector, Br‐IPM, is released. This study assessed the cytotoxic activity of evofosfamide in vitro and its antitumor activity against osteolytic breast cancer either alone or in combination with paclitaxel in vivo. A panel of human breast cancer cell lines were treated with evofosfamide under hypoxia and assessed for cell viability. Osteolytic MDA‐MB‐231‐TXSA cells were transplanted into the mammary fat pad, or into tibiae of mice, allowed to establish and treated with evofosfamide, paclitaxel, or both. Tumor burden was monitored using bioluminescence, and cancer‐induced bone destruction was measured using micro‐CT. In vitro, evofosfamide was selectively cytotoxic under hypoxic conditions. In vivo evofosfamide was tumor suppressive as a single agent and cooperated with paclitaxel to reduce mammary tumor growth. Breast cancer cells transplanted into the tibiae of mice developed osteolytic lesions. In contrast, treatment with evofosfamide or paclitaxel resulted in a significant delay in tumor growth and an overall reduction in tumor burden in bone, whereas combined treatment resulted in a significantly greater reduction in tumor burden in the tibia of mice. Evofosfamide cooperates with paclitaxel and exhibits potent tumor suppressive activity against breast cancer growth in the mammary gland and in bone.

## Introduction

Breast cancer is the most common cancer and the leading cause of cancer mortality in women worldwide 1. Bone metastasis occurs in over 75% of patients with breast cancer and is associated with extensive bone destruction, leading to pathological fractures, bone pain, spinal cord compressions, and hypercalcemia 2. While much progress has been made in the diagnosis and treatment of primary disease, metastatic breast cancer remains a challenging condition to treat and patients with advanced disease continue to die due to metastatic burden. Therefore, there is a great need to identify more effective anticancer therapeutics to improve patients’ disease‐free survival, especially in patients with advanced or metastatic disease.

Solid tumors, including breast cancer, are less well‐oxygenated than the normal tissues from which they arise. This so‐called tumor hypoxia is described by a significant portion of the tumor mass, usually the center of the tumor, becoming less oxygenated due to its long distance from blood vessels and the slower rate of proliferation of the cancer cells in that area 3, 4. This makes cancer cells residing in the hypoxic regions most likely to be resistant to chemotherapy and radiotherapy which then allows the tumor to recur and metastasize. Hence, tumor hypoxia is a major cause of treatment failure, poor outcomes, and recurrence for a variety of malignancies.

These low oxygen levels found in tumor subregions are rarely observed in normal tissues. Tumor hypoxia can therefore serve as the basis for selective cancer therapy, and there are a variety of therapeutic strategies being pursued for the selective targeting of hypoxic tumor cells. Hypoxia‐activated prodrugs (HAPs) are exciting new therapeutics that enable the selective delivery of cytotoxic or cytostatic agents to hypoxic tumor cells. Evofosfamide (formally known as TH‐302) is a HAP composed of 2‐nitroimidazole conjugated to bromo‐isophosphoramide mustard (Br‐IPM) 5. The 2‐nitroimidazole component of evofosfamide acts as an oxygen sensor, causing evofosfamide to fragment in a one electron reductase‐dependent process selectively under hypoxic conditions, releasing the bisalkylating effector, bromo‐isophosphoramide (Br‐IPM) which is a potent alkylating agent creating DNA cross‐links. The DNA damage is recognized by the DNA damage response (DDR). The variant histone H2AX is phosphorylated, producing *γ*H2AX and activating Chk1 and cell cycle arrest 6. In addition, recent studies have shown that under hypoxic conditions, evofosfamide causes the inactivation of thioredoxin reductase 7, which regulates several cellular processes including the protection from damage caused by reactive oxidant species (ROS) and apoptosis by the apoptosis signal‐regulating kinase 1 enzyme (ASK‐1) 8.

Evofosfamide is currently in various stages of clinical trials, due to its efficacy against numerous cancer types including pancreatic cancer, multiple myeloma, soft‐tissue sarcoma, and melanoma 9, 10. However, to date, there has been no investigation looking at the anticancer efficacy of evofosfamide for the treatment of breast cancer at the primary site and, in particular, its growth in bone. It must be noted that the bone marrow itself is hypoxic, when compared with other tissues and metastatic cancer cells such as breast cancer cells, preferentially seed in these hypoxic niches 11. Unlike soft‐tissue tumors, cancer cells in bone adapt to survive in this hypoxic bone microenvironment.

Therefore, the ability to target bone metastases in this hypoxic environment is an important advantage that evofosfamide has over other cancer treatments. Taking this into consideration and the fact that conventional chemotherapeutics are usually cytotoxic to normal cells in the bone marrow, combination of chemotherapeutic agents with evofosfamide should reduce toxicity to these bone cells, especially since we have recently shown evofosfamide to be nontoxic to normal bone cells 12. This study assessed the cytotoxic activity of evofosfamide against a panel of human breast cancer cell lines in vitro and evaluated its antitumor efficacy alone and in combination with the chemotherapeutic agent paclitaxel in vivo, using animal models of osteolytic breast cancer.

## Materials and Methods

### Cells

The human breast cancer cell lines MDA‐MB‐468, MDA‐MB‐453, MCF‐7, T47D, and ZR‐75 and the breast epithelial cell lines MCF‐10A and MCF‐12A were obtained from ATCC (Manassas, VA). The MDA‐MB‐231 derivative cell line, MDA‐MB‐231‐TXSA, was kindly provided by Dr. Toshiyuki Yoneda (formerly at University of Texas Health Sciences Centre, San Antonio, TX). The generation of luciferase‐tagged MDA‐MB‐231‐TXSA‐TGL was described previously 13. Normal mammary fibroblast cells were provided by Wendy Ingman (University of Adelaide, Australia), and the dermal fibroblasts were a gift from John E.Greenwood (Adult Burn Centre, Royal Adelaide Hospital, Australia). The breast cancer lines and fibroblasts were cultured in Dulbecco's Modified Eagle's Medium (DMEM), supplemented with 2 mmol/L glutamine, 100 IU/mL penicillin, 160 *μ*g/mL gentamicin, and 10% fetal bovine serum (Life Technologies, Carlsbad, CA). The epithelial cell lines were cultured in membrane epithelial basal media (Lonza, Basel, Switzerland) containing 10% fetal bovine serum. All cell lines were maintained in a 5% CO_2_‐containing humidified atmosphere.

### Drugs

Evofosfamide was provided by Threshold Pharmaceuticals (South San Francisco, CA) and dissolved in sterile saline at a concentration of 13.2 mmol/L. The ZVAD‐fmk (Caspase Inhibitor‐1) was purchased from Calbiochem (Inc. La Jolla, CA), and Paclitaxel was purchased from Ebewe Pharma (A‐4866; Unterach, Austria).

### Cell viability assay

**Figure 1:**
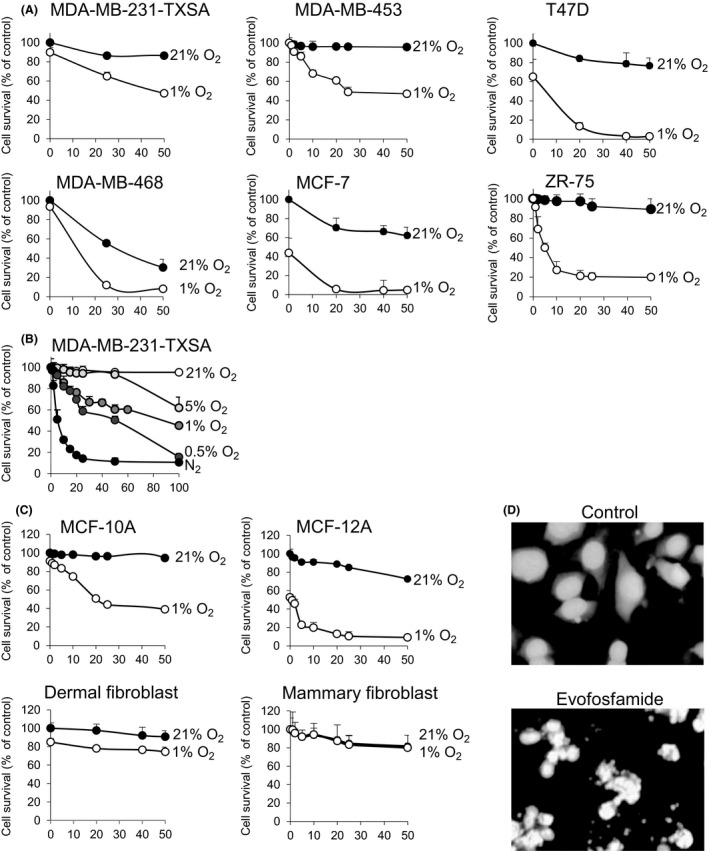
Activity of evofosfamide against breast cancer, epithelial cells, and fibroblasts in vitro. (A) Six breast cancer cell lines were seeded in 96‐well plates at 1 × 10^4^ cells per well and treated with increasing doses of evofosfamide in normoxic (21% O_2_) and hypoxic (1% O_2_) conditions for 48 h. (B) The breast cancer cell line MDA‐MB‐231‐TXSA was treated with an increasing dose of evofosfamide under normoxic (21% O_2_) and in various hypoxic conditions from 0% to 5% O_2_ for 24 h. (C) Evofosfamide reduced cell viability in both epithelial breast cell lines MCF‐10A and MCF‐12A selectively under hypoxic conditions (1% O_2_) 48 h after treatment. Dermal and mammary fibroblasts were relatively resistant to evofosfamide under the same conditions. Cell viability of all cell lines was assessed by crystal violet staining. (D) DAPI nuclear fluorescence stain of untreated MDA‐MB‐231‐TXSA cells showing nuclei homogenously stained. Cells treated with 50 μmol/L evofosfamide under hypoxic conditions for 24 h exhibit changes in the nuclei consistent with the induction of apoptosis. Data points show means of quadruplicate results from a representative experiment, repeated at least twice and presented as the mean ± SD of quadruplicate wells and expressed as a percentage of the number of control cells.

To determine the cytotoxic effects of evofosfamide on cell growth, 1 × 10^4^ cells per well were seeded in 96‐well microtiter plates and allowed to adhere overnight. Cells were then treated with increasing concentrations of evofosfamide (1–50 *μ*mol/L) for 48 h as shown in Figure 1A or 24 h (1–100 *μ*mol/L) as shown in Figure 1B, under normoxic and various hypoxic conditions. Cell viability was determined by crystal violet staining, and optical density was measured at 570‐nm wavelength (OD570). Experiments were performed in triplicate and repeated at least 3 times. Results of representative experiments are presented as the mean ± SD.

### Apoptosis analysis

#### DAPI staining of nuclei

Cells were seeded on plastic chamber slides and treated at 1% O_2_ with evofosfamide at 50 *μ*mol/L for 24 h. After two washes with PBS, cells were fixed in ethanol:acetic acid (6:1) for 10 min, washed again with PBS, and incubated with 0.8 mg/mL of 4′,6‐diamidine‐2′‐phenylindole dihydrochloride (DAPI; Roche Diagnostics, Mannheim, Germany) in methanol for 15 min. After several washes in PBS, the coverslips were mounted on ProLong^®^Gold antifade (Life Technologies). DAPI staining was visualized by fluorescence microscopy.

#### Measurement of DEVD‐caspase activity

DEVD‐caspase activity was assayed by cleavage of zDEVD‐AFC, a fluorogenic substrate based on the peptide sequence at the caspase‐3 cleavage site of poly (ADP‐ribose) polymerase. Cells (1 × 10^4^/well) grown in 96‐well plates were treated as indicated, washed once with PBS, and resuspended in 30 *μ*L lysis buffer containing 5 mmol/L Tris‐HCl, 5 mmol/L EDTA, and 10% Igepal (pH 7.5). Cell lysate (15–20 *μ*g of protein) was added to each assay tube containing 8 *μ*mol/L substrate in 1 mL fluorometric protease buffer (50 mmol/L HEPES, 10% sucrose, 10 mmol/L DTT, 0.1% CHAPS [pH 7.4]). After 4–5 h at room temperature, fluorescence was quantified (Ex 400 and Em 505) in a Perkin‐Elmer LS50 fluorescence spectrometer. Results were expressed relative to the protein concentration of the sample and determined using a commercial BCA protein assay reagent from Thermo Fisher Scientific (Waltham, MA).

### Western blot analysis

Cells were treated with evofosfamide at 50 *μ*mol/L in a time‐dependent manner (0, 6, 12, 24, 48 h) under normoxic (21% O_2_) and hypoxic (1% O_2_) conditions and lysed in buffer containing 10 mmol/L Tris‐HCl, pH 7.6, 150 mmol/L NaCl, 1% Triton X‐100, 0.1% sodium dodecyl sulfate (SDS), 2 mmol/L sodium vanadate, and a protease inhibitor cocktail (Roche Diagnostics). Protein lysates were heated at 70°C for 10 min and loaded into 4–12% polyacrylamide gels for electrophoresis under reducing conditions. Separated proteins were electrophoretically transferred to PVDF membranes (GE Healthcare, Buckinghamshire, UK) and blocked in PBS containing 5% blocking reagent (GE Healthcare) for 1 h at room temperature.

Immunodetection was performed overnight at 4°C in PBS/blocking reagent containing 0.1% Tween 20, using the following primary antibodies at the dilutions suggested by the manufacturer. mAb anticaspase‐8, mAb anticaspase‐3, pAb antibid, pAb anticaspase‐9, pAb PI3 kinase Class III, pAb phospho‐p44/42 MAPK, pAb p44/42 MAPK, pAb phospho‐Akt, and pAb Akt were purchased from Cell Signaling Technology (Beverly, MA), pAb anti‐inhibitor of apoptosis 1 (cIAP1), pAb anti‐inhibitor of apoptosis 2 (cIAP2), pAb anti‐XIAP from R&D systems, pAb anti‐BAX, pAb anti‐p53, pAb anti‐p21, and mAb anti‐Bcl‐2 were purchased from Santa Cruz Biotechnology (Dallas, Texas) and pAb anti‐poly‐(ADP‐Ribose) polymerase (PARP) from Roche Diagnostics. Anti‐actin mAb (Sigma, Saint Louis, MO) was used as a loading control. Membranes were then rinsed several times with PBS containing 0.1% Tween‐20 and incubated with 1:5000 dilution of anti‐mouse, anti‐goat, or anti‐rabbit alkaline phosphatase‐conjugated secondary antibodies (Thermo Fisher Scientific) for 1 h. Visualization and quantification of protein bands was performed using the ECF substrate reagent kit (GE Healthcare) on a FluorImager (Molecular Dynamics Inc., Sunnyvale, CA).

### Animals

Female athymic mice at 5 weeks old (Institute of Medical and Veterinary Services Division, Gilles Plains, SA, Australia) were acclimatized to the animal housing facility for a minimum period of 1 week prior to the commencement of experimentation. The general physical well‐being and weight of animals were monitored continuously throughout the experiments. All mice were housed under pathogen‐free conditions, and all experimental procedures on animals were carried out with strict adherence to the rules and guidelines for the ethical use of animals in research and were approved by the Animal Ethics Committees of the University of Adelaide and the Institute of Medical and Veterinary Science, Adelaide, SA, Australia.

### Mammary fat pad injections of breast cancer cells

MDA‐MB‐231‐TXSA‐TGL human breast cancer cells were cultured as described above until they reached 70–80% confluency. Cells were removed from flasks with 2 mmol/L EDTA and resuspended in 1 × PBS at 1 × 10^5^ cells/10 *μ*L and kept on ice in an eppendorf tube. An equal volume of Matrigel^™^‐HC (BD Biosciences, Bedford, MA) was added to the cells and resuspended. The mice were anesthetized by Isoflurane (Faulding Pharmaceuticals, SA, Australia), the mammary fat pad area of the mice was wiped with ethanol, and the skin was lifted over the left outermost nipple. Finally, 20 *μ*L of cells was injected into the mammary fat pad using a 25‐gauge needle, and animals were allowed to recover under a heat lamp before being transferred into cages. Mice were then assigned randomly into groups of 10 animals each. Evofosfamide was given via intraperitoneal injection at a dose of 50 mg/kg once a day for 5 consecutive days, followed by 2 days rest. Paclitaxel was given at a dose of 6.25 mg/kg subcutaneously once a week. The dosing started 7 days after cancer cell implantation and was given every week until the end of the experiment, at day 21.

### Intratibial injections of breast cancer cells

MDA‐MB‐231‐TXSA‐TGL cells were cultured as described above. The left tibia was wiped with 70% ethanol, and a 27‐gauge needle coupled to a Hamilton syringe was inserted through the tibial plateau with the knee flexed; 1 × 10^5^ MDA‐MB‐231‐TXSA‐TGL cells resuspended in 10 *μ*L of PBS were then injected in the marrow space. All animals were injected with PBS in the contralateral tibia as the control. Mice were then assigned randomly into groups of 10 animals per group and received the same treatments described in the mammary fat pad model.

### In vivo bioluminescent imaging

Noninvasive, whole‐body imaging to monitor luciferase‐expressing MDA‐MB‐231‐TXSA‐TGL cells in mice was performed weekly using the IVIS 100 Imaging system (Xenogen, Alameda, CA). Mice were injected i.p. with 100 *μ*L of the d‐Luciferin solution at final dose of 3 mg/20 g mouse body weight (Xenogen) and then gas anesthetized with Isoflurane (Faulding Pharmaceuticals). Images were acquired for 0.5–30 sec (representative images are shown at 1 sec), and the photon emission transmitted from mice was captured and quantitated in photons/sec/cm^2^/sr using Xenogen Living image (Igor Pro version 2.5) software.

### Microcomputed tomography ex vivo analysis

Limbs for *μ*CT analysis were surgically resected and scanned using the SkyScan‐1072 high‐resolution *μ*CT Scanner (Skyscan, Kontich, Belgium). The Scanner was operated at 80 kV, 120 *μ*A, rotation step 0.675, 0.5 mm Al filter and scan resolution of 5.2 *μ*m/pixel. The cross‐sections were reconstructed using a cone‐beam algorithm (software Cone rec, Skyscan). Using the 2D images obtained from the *μ*CT scan, the growth plate was identified and 400 sections were selected starting from the growth plate/tibial interface and moving down the tibia. For quantification, 3D evaluation was performed on all data sets acquired by selecting total bone of the proximal tibia, to determine 3D bone morphometric parameters (software CTAn, Skyscan). The cross‐sections were reconstructed using a cone‐beam algorithm (software Cone_rec, Skyscan). Files were then imported into CTAn software (Skyscan) for 3D analysis and 3D image generation. All images are viewed and edited using ANT visualization software (Skyscan).

### Histology

Tibiae were fixed in 10% (v/v) buffered formalin (24 h at 4°C), followed by 2 weeks of decalcification in 0.5 mol/L EDTA/0.5% paraformaldehyde in PBS, pH 8.0 at 4°C. Complete decalcification was confirmed by radiography and tibiae were then paraffin embedded. Five‐micron longitudinal sections were prepared and stained with H&E. Analysis was performed using the Nanozoomer and the Hamamatsu software.

### Data analysis and statistics

Experiments were performed in triplicate, and data presented as mean ± SE. All statistical analysis was performed using SigmaStat for Windows version 3.0 (Systat Software, Inc., Port Richmond, CA) using the unpaired Students’ t*‐*test. Measures of association between two variables were assessed using the Spearman's rank correlation coefficient. Comparisons between groups were assessed using a one‐way analysis of variance test. In all cases, *P* < 0.05 was considered statistically significant.

## Results

### Evofosfamide exhibits hypoxia‐selective cytotoxicity on human breast cancer cells

A panel of human breast cancer cell lines (MDA‐MB‐231‐TXSA, MDA‐MB‐453, MDA‐MB‐468, ZR‐75, MCF‐7, and T47D) were assessed for their sensitivity to the cytotoxic effects of evofosfamide under normoxic (21% O_2_) and hypoxic (1% O_2_) conditions. Evofosfamide exhibited relatively minimal toxicity under normoxic conditions, whereas under hypoxic conditions (1% O_2_), evofosfamide reduced cell viability in a dose‐dependent manner after 48 h of treatment, with IC_50_ values ranging between 1 and 25 *μ*mol/L and a maximum of 50–90% loss of viability at the highest dose of 50 *μ*mol/L (Fig. 1A). The hypoxia selectivity of evofosfamide, in the MDA‐MB‐231‐TXSA cells measured as a ratio of IC_50_ values under varying conditions of hypoxia (0%, 0.5%, 1.0%, and 5.0% oxygen), was greater than 200‐fold during extreme hypoxia of <1.0% after 24 h of treatment (Fig. 1B). Of interest, evofosfamide was also cytotoxic to MCF‐10A and MCF‐12A cells lines, normally regarded as normal epithelial cell lines of breast origin, when subjected under the same hypoxic conditions as the breast cancer cell lines, with IC_50_ values of 25 *μ*mol/L and 2 *μ*mol/L, respectively. In contrast normal human dermal fibroblasts and human primary mammary fibroblasts were relatively resistant to the cytotoxic activity of evofosfamide, with IC_50_ values of >50 *μ*mol/L and 40 *μ*mol/L, respectively (Fig. 1C). Evofosfamide‐mediated cytotoxicity in the breast cancer cell line MDA‐MB‐231‐TXSA under hypoxic conditions was associated with morphological changes characteristic of apoptosis, including chromatin condensation, nuclear fragmentation as assessed by DAPI staining of nuclei (Fig. 1D).

### Caspase activation is secondary in the apoptotic activity of evofosfamide

**Figure 2:**
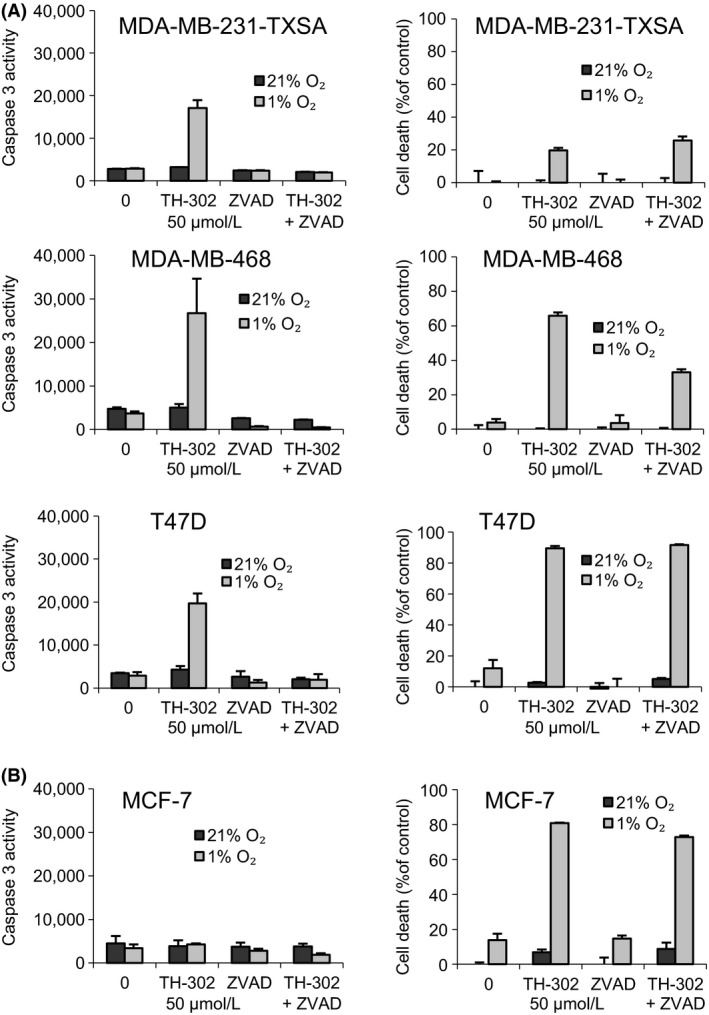
Evofosfamide‐induced apoptosis in breast cancer cells in vitro. (A) Four breast cancer cell lines were seeded in 96‐well plates at 1 × 10^4^ cells per well and treated with evofosfamideat 50 μmol/L or coincubated with the broad specificity caspase inhibitor z‐VAD‐fmk (50 μmol/L). To exclude possible toxic effects of the inhibitor, cells were also treated with the inhibitor alone under normoxic and hypoxic (1% O_2_) conditions. Cell lysates were used to determine caspase‐3‐like activity, using the caspase‐3‐specific fluorogenic substrate, zDEVD‐AFC, and cell viability was assessed via crystal violet staining. Data points show means of quadruplicate results from a representative experiment, repeated at least twice; bars ± SD.

The observed decrease in cell viability with evofosfamide treatment under hypoxic conditions was associated with morphological changes characteristic of apoptosis, including chromatin condensation and nuclear fragmentation, concomitant with an increase in caspase‐3 activation. However, coadministration with the pan‐caspase inhibitor ZVAD‐fmk failed to prevent the cytotoxic activity of evofosfamide under hypoxic conditions, despite irreversibly inhibiting caspase‐3 activity (Fig. 2A) suggesting that the mechanisms of evofosfamide‐mediated cytotoxicity are in part caspase independent. This is also supported by the observation that evofosfamide under hypoxic conditions was potently cytotoxic against MCF‐7 cells which are caspase‐3 deficient. 14 (Fig. 2B).

**Figure 3:**
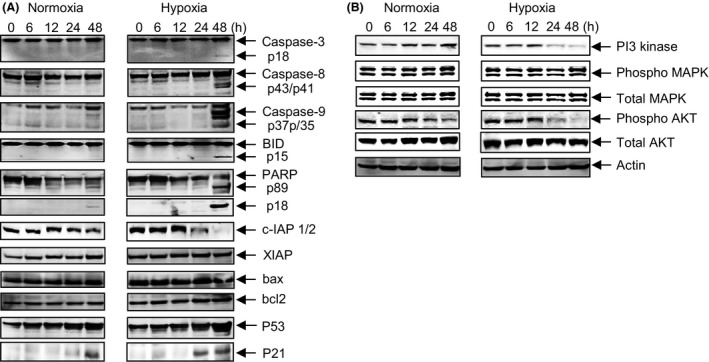
Apoptotic signaling of evofosfamide as a single agent against MDA‐MB‐231‐TXSA cells. (A) MDA‐MB‐231‐TXSA‐TGL cells were seeded at 2 × 10^6^ per T25 flask and were treated with evofosfamide at 50 μmol/L under normoxic (21% O_2_) and hypoxic (1% O_2_) conditions. Cells were then lysed and protein lysates were collected at 0, 6, 12, 24, and 48 h after treatment. Cell lysates were analyzed by polyacrylamide gel electrophoresis and transferred to PVDF membranes for immunodetection as described in Materials and Methods section. Evofosfamide treatment resulted in cleavage and prominent activation of the initiator caspase‐8 followed by caspase‐9, and caspase‐3 and the concomitant processing of BID and PARP protein, only at the later time point of 48 h, indicating that these changes were an effect rather than a cause of evofosfamide‐induced apoptosis. cIAP1/2 levels were significantly reduced with evofosfamide treatment, under hypoxic conditions at the 24‐ and 48‐h time points, whereas the levels of XIAP, BAX, and Bcl‐2 remained unchanged. (B) A robust reduction in PI3 kinase and phosphorylated AKT under hypoxic conditions after treatment with TH‐302 at 24 and 48 h. However, no significant changes in the levels of either phosphorylated or total MAPK were detected following treatment.

As a prelude to testing the anticancer efficacy of evofosfamide in vivo*,* the MDA‐MB‐231‐TXSA cell line was chosen to further characterize the molecular determinants involved in apoptotic signaling, mediated by evofosfamide (Fig. 3A). Under hypoxic conditions (1% O_2_), evofosfamide treatment resulted in the prominent activation of the caspase cascade with robust cleavage of the initiator caspase‐8 followed by caspase‐9, and caspase‐3 and the concomitant processing of the mitochondrial proapoptotic Bcl‐2 family protein BID and the poly ADP‐ribose polymerase (PARP) protein. While these changes were observed under hypoxic conditions, nonetheless this occurred only at the later time point of 48 h after the onset of cell death, indicating that these changes were an effect rather than a cause of evofosfamide‐induced apoptosis. The levels of inhibitor of apoptosis proteins 1 and 2 (cIAP1/2) under hypoxic conditions were significantly reduced with evofosfamide treatment, which occurred at the 24‐ and 48‐h time points, whereas the levels of XIAP, BAX, and Bcl‐2 remained unchanged. Due to the DNA alkalization caused by the Br‐IPM component of evofosfamide, upregulation of p53 was observed after 48 h of evofosfamide exposure in both normoxic and hypoxic conditions, leading to the upregulation of the p53 target protein p21 after 48 h in normoxia and after 24 h in hypoxia. Additional signaling pathway analysis demonstrated a robust reduction in PI3 kinase and inhibition of phosphorylated AKT which was evident at 24 and 48 h after treatment with evofosfamide. However, no significant changes in the levels of either phosphorylated or total MAPK were detected following treatment.

### Effect of evofosfamide and paclitaxel on the growth of orthotopic breast cancer xenografts

**Figure 4:**
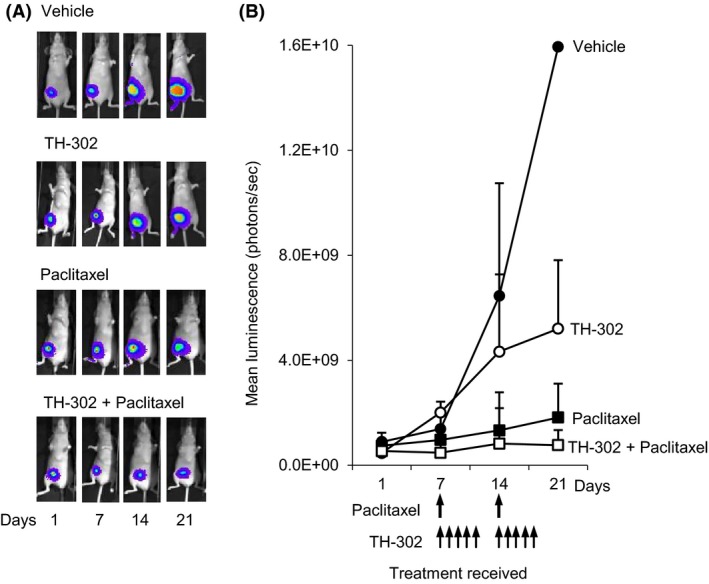
Paclitaxel cooperates with evofosfamide to reduce MDA‐MB‐231‐TXSA‐TGL orthotopic tumors in vivo. Mice were treated as described in Materials and Methods section and imaged weekly using the Xenogen IVIS 100 bioluminescence imaging system. (A) Representative whole body bioluminescent images of the mammary tumors of a single animal from each group and (B) the line graph, showing average tumor signal over time, expressed as mean photon counts per second during the course of the experiments are shown. Animals receiving treatment with evofosfamide and paclitaxel as single agents showed a significant delay in tumor growth. In addition, all mice receiving the combination of evofosfamide and paclitaxel showed a further delay of tumor growth when compared with each agent individually. Data shown in each case are the average bioluminescent imaging from all animals in that group: points are means ± SEM.

To investigate the anticancer efficacy of evofosfamide, paclitaxel ,and the combination of both against tumors growing in the orthotopic site, luciferase‐tagged MDA‐MB‐231‐TXSA cells were injected directly into the mammary fat pad of athymic female nude mice and treatment was initiated 7 days post cancer cell transplantation. These cells form aggressive, rapidly growing tumors when injected into the mammary fat pad, which can be accurately monitored and quantified using noninvasive bioluminescence imaging 13. Animals treated with vehicle showed an exponential increase of mean photon emission, associated with an increase in tumor burden, which was evident from day 7 onwards, reaching a maximum signal at day 21, at which point animals had to be humanely killed. In contrast, all animals treated with evofosfamide or paclitaxel as single agents showed a significant reduction in tumor burden over the same period, whereas the combination of both was highly effective which completely prevented tumor growth (Fig. 4A and B).

### Effect of evofosfamide and paclitaxel against breast cancer‐induced bone destruction

**Figure 5:**
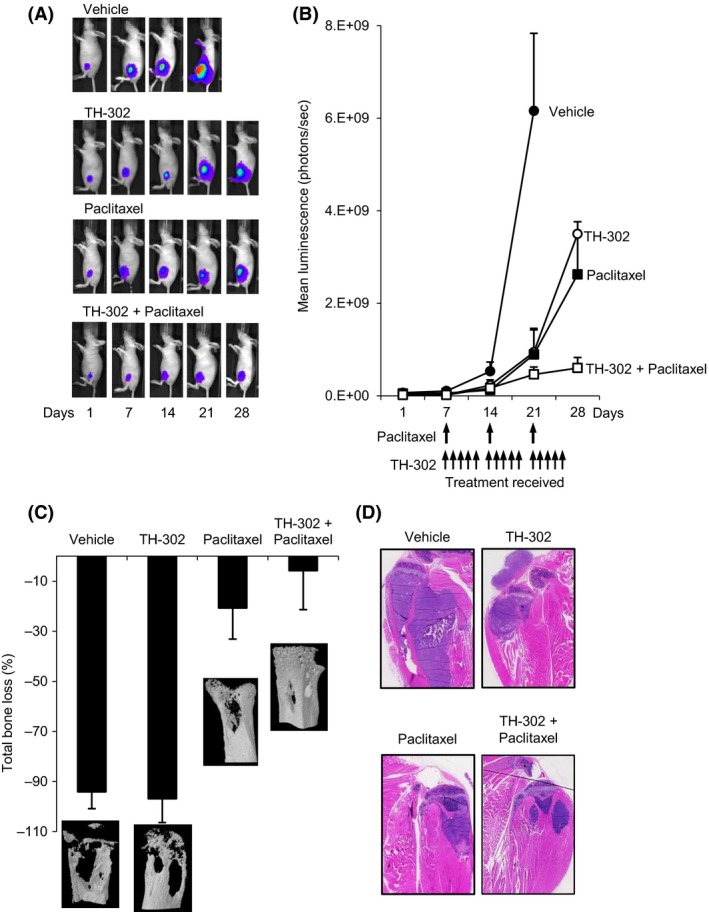
Paclitaxel cooperates with evofosfamide for increased anticancer efficacy against the breast cancer line MDA‐MB‐231‐TXSA‐TGL in vivo. MDA‐MB‐231‐TXSA‐TGL cells were injected directly into the tibial marrow cavity of 4 week female athymic mice, allowed to establish for 7 days, as described in Materials and Methods section, mice were imaged weekly using the Xenogen IVIS 100 bioluminescence imaging system. (A) Representative whole‐body bioluminescent images of a single mouse from each group during the course of the experiment are shown. (B) The line graph represents average tumor signal over time, measured as photon counts per second. Evofosfamide and paclitaxel as single agents reduced tumor growth, and in addition, the combination of both resulted in an even greater inhibition of tumor growth. (C) Quantitative assessment of total bone loss (%) comparing the tumor‐bearing tibiae of each group to the contralateral tibiae and the qualitative 3‐D micro CT images show the osteolytic nature of the MDA‐MB‐231‐TXSA‐TGL cell line, which was reduced by paclitaxel and the combination of evofosfamide and paclitaxel. (D) Representative H&E‐stained tibial sections from mice showing an inhibition of intra‐ and extramedullary growth with evofosfamide, paclitaxel, and the combination of both.

To evaluate the activity of evofosfamide against tumor growth in bone and its effects on cancer‐induced bone destruction, a xenogeneic tumor model was used, in which the MDA‐MB‐231‐TXSA‐TGL cells were transplanted directly into the tibial marrow cavity of athymic mice. All vehicle‐treated animals showed an exponential increase of mean photon emission associated with an increase in tumor burden, which was clearly evident from day 14 onwards. By day 21, all animals developed large intratibial tumors that penetrated the cortical bone with the tumor mass, invading the surrounding soft tissue. For ethical reasons, all vehicle‐treated animals were humanely killed on day 21 due to high tumor load and extensive osteolysis. In contrast, animals treated with evofosfamide or paclitaxel as single agents showed a significant delay in tumor growth and an overall reduction in tumor burden, whereas the combination of both agents demonstrated a far superior anticancer efficacy in bone (Fig. 5A and B).

At the end of the experiment, the tibiae of all mice were dissected and assessed, using high‐resolution *μ*CT for both quantitative and qualitative analysis of bone destruction (Fig. 5C). Extensive osteolysis was clearly evident in the vehicle‐treated animals such that the net loss in bone volume (BV) was 94% in the left tumor‐bearing tibiae when compared with the contralateral nontumor‐bearing tibiae. While evofosfamide treatment reduced tumor burden, this did not translate to prevention of bone destruction such that the extent of osteolysis was not significantly different than the vehicle‐treated group. In contrast, treatment with paclitaxel alone was significantly effective, decreasing bone loss to 21%. Combination therapy with evofosfamide and paclitaxel demonstrated additional protection of the bone architecture such that the net loss of BV was only 6%, although this improvement in bone protection by the combination therapy was not statistically significant when compared with paclitaxel alone. Histological examination of representative sections of the tibial sections show the MDA‐MB‐231‐TXSA‐TGL tumor growing within the marrow cavity and in the surrounding tissue (Fig. 5D). Trabecular and cortical bone destruction was also evident, confirming the micro‐CT data. Treatment with evofosfamide, paclitaxel, and the combination of both significantly reduced tumor size in the bone marrow confirming the bioluminescence data but did not completely eliminate breast cancer growth in bone.

## Discussion

Tumor hypoxia is a major cause of treatment failure and poor outcome for a wide variety of malignancies 15. Within most solid tumors, there are significant areas of hypoxia, which contain cancer cells that resist conventional anticancer chemotherapy and radiotherapy, and this predisposes to tumor recurrence and metastasis 16, 17. However, tumor hypoxia also offers treatment opportunities, exemplified by the development of highly active compounds that can specifically target tumor hypoxic zones. In this study, we assessed the hypoxia‐selective cytotoxicity of evofosfamide against a panel of human breast cancer cell lines in vitro and investigated the anticancer efficacy of evofosfamide as monotherapy and in combination with paclitaxel against breast cancer growing in the orthotopic site of the mammary gland and in the bone marrow using the aggressive and highly osteolytic MDA‐MB‐231‐TXSA breast cancer cells. Consistent with our previous published data 12, evofosfamide exhibited relatively minimal toxicity under normoxic conditions, whereas under increasing hypoxia evofosfamide treatment of a variety of human breast cancer cell lines dose dependently decreased cell viability. Normal breast epithelial cell lines MCF‐10A and MCF‐12A were also equally sensitive to evofosfamide under hypoxic conditions, which may be related to their proliferative capacity being similar to that of breast cancer cells. In contrast, primary dermal fibroblasts and normal mammary fibroblasts were relatively resistant to the cytotoxic activity of evofosfamide under the same conditions.

It is well established that the clinical management of breast cancer patients with different amplifications of markers such as ER+, PR+, and HER2 indicates different therapeutic strategies. Our results do not show any direct correlation between these markers and the cytotoxic activity of evofosfamide, suggesting perhaps a universally effective response. However, recent studies using a limited number of cell lines have shown a link between BRCA‐1, triple negativity of breast cancer and its response to evofosfamide 18. In this context, an extensive investigation using a large cohort of breast cancer cell lines and patient data will be required to delineate such differences in evofosfamide therapeutic response.

While evofosfamide treatment induced changes characteristic of apoptosis induction, coaddition with the broad‐spectrum caspase inhibitor zVAD‐fmk failed to protect breast cancer cells from the cytotoxic activity of evofosfamide under hypoxia, suggesting the involvement of caspase independent mechanism. This is also reflected by the activation of caspase‐3, caspase‐8, caspase‐9, cleavage of the Bcl‐2 family protein Bid, and the downregulation of c‐IAP1/2, which occurred 48 h post evofosfamide treatment, well after apoptosis induction and therefore, likely to represent events of the cell death process rather than a cause of evofosfamide‐induced apoptosis. When evofosfamide is activated under hypoxia, the cytotoxic compound Br‐IPM induces 1′3′‐cross‐linkage of DNA, which results in S139 phosphorylation of the histone H2AX leading to cell death 19. This DNA damage is often associated with caspase independent mechanisms as previously reported 20 and is reflected by PARP cleavage leading to the upregulation of p53 followed by upregulation of the p53 target protein and cell cycle regulating gene p21 as clearly observed here.

The therapeutic advantage of evofosfamide is expected to be greatest in combination with adjuvant cytotoxic chemotherapy. In this context, we tested the activity of evofosfamide in combination with paclitaxel, in a preclinical model of breast cancer development and progression. We selected the breast cancer cell line MDA‐MB‐231‐TXSA, which we have shown in vitro to be the least sensitive to evofosfamide, such that any additive or synergistic activity will be readily detected. The antitumor effect of evofosfamide as a single agent in the mammary gland was most prominent from day 14 onwards when tumor size was approaching 1.0 cm^3^ which coincided with the presence of hypoxic tumor cores seen when tumors were excised from parallel untreated animals and assessed histologically at the same time (data not shown). While Paclitaxel as a single agent was highly effective in reducing tumor burden, the combination of both completely prevented growth of the tumor within the mammary gland.

MDA‐MB‐231‐TXSA cells are highly osteolytic and when transplanted in to the tibial marrow cavity of mice lead to extensive bone destruction 13, 21. This in vivo model mimics the late stages of bone metastasis and is ideally suited for monitoring the effects of drug treatment on breast cancer growth in the bone and also on cancer‐induced bone destruction 13. To date, the activity of evofosfamide against breast cancer growing in bone has not been reported. When breast cancer cells were injected intratibially, evofosfamide treatment inhibited tumor growth in bone, leading to a significant reduction in the overall tumor burden. However, the reduction in tumor burden did not translate to a significant inhibition of osteolysis attesting to the aggressive osteolytic properties of these cells. In contrast, paclitaxel as a single agent was highly effective in reducing tumor load in bone while also protecting the bone from cancer‐induced bone destruction. The combined treatment resulted in a reduction in tumor burden.

It is important to note that while hematological toxicity with evofosfamide treatment has been minimal, to date no data exist on the effects of evofosfamide or indeed any other HAP, on normal bone metabolism in the context of osteoclasts, osteoblasts, or osteocyte survival and function. We have compared the micro architectural bone morphometric parameters of the contralateral nontumor injected tibiae from untreated and evofosfamide‐treated animals and showed no obvious histological abnormalities on bone parameters including the number and viability of osteoclasts, or osteoblasts, per surface area (data not shown), whereas high‐resolution micro‐CT analysis demonstrated no changes in micro architectural bone parameters, such as total or trabecular BV measurements with evofosfamide treatment (data not shown). These results are consistent with our previously published data in this context using a model of osteosarcoma 12.

Evofosfamide is currently being evaluated in numerous phase III and phase II clinical trials against a variety of cancer types either as monotherapy or in combination with conventional chemotherapy with promising results. Our data further support the clinical development of evofosfamide as a novel approach in the treatment of patients with breast cancer, especially those with existing bone metastases.

## Conflict of Interest

There are no financial disclosures from any of the authors in this article.
